# Hemophagocytic Lymphohistiocytosis as Initial Presentation of Malignancy in Pediatric Patients: Rare but Not to Be Ignored

**DOI:** 10.3390/children8121083

**Published:** 2021-11-24

**Authors:** Hye-ji Han, Kyung Taek Hong, Hyun Jin Park, Bo Kyung Kim, Hong Yul An, Jung Yoon Choi, Hyoung Jin Kang

**Affiliations:** 1 Departments of Pediatrics, Seoul National University College of Medicine, Seoul 03080, Korea; haeji1993@gmail.com (H.-j.H.); fionajin05@snu.ac.kr (H.J.P.); imkbk@snuh.org (B.K.K.); anongg@snu.ac.kr (H.Y.A.); choijy@snu.ac.kr (J.Y.C.); kanghj@snu.ac.kr (H.J.K.); 2 Cancer Research Institute, Seoul National University, Seoul 03080, Korea; 3 Wide River Institute of Immunology, Hongcheon 25159, Korea

**Keywords:** hemophagocytic lymphohistiocytosis, malignancy-triggered HLH, HScore, pediatric HLH, secondary HLH

## Abstract

It is complicated to establish a consensus on the management and diagnosis of malignancy-triggered hemophagocytic lymphohistiocytosis (M-HLH) in children, as an initial presentation of malignancy is complicated. In this paper, we analyze the clinical characteristics and outcomes of eight pediatric patients in which M-HLH was the initial presentation of malignancy. All patients had hematologic malignancies: three subcutaneous panniculitis-like T-cell lymphomas, two acute lymphoblastic leukemias, two anaplastic large cell lymphomas, and a systemic EBV + T-cell lymphoma of childhood. The incidence rate of M-HLH among leukemia and malignant lymphoma patients in our institution was 1.9%. From the initial diagnosis of HLH, the median time taken to be diagnosed as a malignancy was about 1.3 months. The majority of patients received HLH-targeted immunosuppression and/or etoposide at first. The patients’ clinical response to treatment for HLH and malignancies were varied. Five out of the eight patients died, one of whom died due to HLH-related cerebral edema after the initiation of chemotherapy. The median overall survival was 1.6 years. In order to improve the survival rate, the early detection of M-HLH, rapid screening for malignancy, and complete control of M-HLH with HLH-directed therapy followed by a thorough response monitoring are required.

## 1. Introduction

Hemophagocytic lymphohistiocytosis (HLH) is a rare disorder caused by hyperinflammation or immune dysregulation. The Histiocyte Society proposed a diagnostic criteria and it was revised for the HLH-2004 criteria (Table 1). The diagnosis requires the fulfillment of five out of eight clinical features, including fever, splenomegaly, bicytopenia, hypofibrinogenemia or hypertriglyceridemia, hyperferritinemia, increased level of soluble CD25, evidence of hemophagocytosis, and decreased NK-cell cytotoxicity [1].

HLH can be separated into two groups: primary HLH and secondary HLH. Whereas malignancy-associated HLH is the most common type of secondary HLH in adults, the relative lack of studies in children makes diagnosis even more complicated [2]. HLH can manifest as an initial presentation during the treatment course in the context of malignancy. Malignancy-triggered HLH (M-HLH) due to the underlying disease itself can be distinguishable from those related with therapy, such as chemotherapy or hematopoietic stem cell transplantation (HSCT). Chemotherapy-triggered HLH and post-HSCT HLH vary from M-HLH in the types of conditions and risks involved when approaching the differential diagnosis before immunosuppressive or cytoreductive therapy [3]. In this article, we only included the cases of M-HLH preceding the diagnosis of underlying malignancy that has rarely reported in children [4].

The appropriate evaluation and management of M-HLH are very important because poorly-managed M-HLH can invariably result in a fatal course [5]. As one of the quantitative tools to evaluate the possibility of HLH and to help differential diagnosis, the HScore was developed for adults (Table 1). The HScore was validated based on a group with a median age of 51 years, and the cutoff value of 169 corresponded to a sensitivity of 93% and a specificity of 86% [6]. However, the appropriate evaluation method for M-HLH in children has not yet been established.

We analyzed the clinical characteristics and outcomes of pediatric M-HLH as an initial presentation by applying the HScore and HLH-2004 criteria. This study aims to investigate the appropriate treatment approach for M-HLH, which is rare in children, but should not be ignored.

## 2. Materials and Methods

This study retrospectively reviewed patients with confirmed diagnosis of malignancy who were initially presented as HLH between 2014 and 2020 at Seoul National University Children’s Hospital. The patients were diagnosed according to the diagnostic criteria established in the HLH-2004 trial. The genetic study for HLH refers to a comprehensive panel and sequencing for UNC13D and PRF1. The panel for familial HLH covers ADA, AP3B1, BLOC1S6, BTK, CD27, FADD, FAS, FASLG, IL2RA, IL2RG, ITK, LVST, MAGT1, MVK, MY05A, PNP, PRF1, RAB27A, RECQL4, SH2D1A, SLC7A7, STX11, STXBP2, UNC13D, WAS, and XIAP genes. The significance of variants was defined according to standards and guidelines of the American College of Medical Genetics and Genomics and the Association for Molecular Pathology [7].

The study was approved by the international review board of Seoul National University Hospital (H 2104-143-1214). Informed consent was waived because it was judged that it was practically impossible to obtain informed consent as the patients had already died or there was no follow-up, and the risk to the patients was extremely low even if consent was waived. The overall and progression-free survival rate was estimated for 1-year interval and survival curves were plotted using the Kaplan–Meier survival analysis. Progression-free survival was defined as the time from the diagnosis of malignancy to disease progression, death from any cause, or last follow-up. Overall survival was defined as the time from the diagnosis of malignancy to death from any cause or last follow-up. The HScore was measured at initial diagnosis, on the 15th day of treatment and just before initiation of chemotherapy for malignancy. Missing data were filled with the recent value within last one month.

## 3. Results

### 3.1. Type of Malignancy

The median age was 9.4 years old (range 5–18 years) at the time of diagnosis of malignancy. Eight patients who presented with HLH as an initial presentation of malignancy were included: subcutaneous panniculitis-like T-cell lymphoma (SpTCL) (*n* = 3); acute lymphoblastic leukemia (ALL) (*n* = 2); anaplastic large cell lymphoma (ALCL) (ALK+ *n* = 2); and systemic EBV + T-cell lymphoma of childhood (EBV T L) (*n* = 1). Among all pediatric patients who were diagnosed as malignancies at our institution, the incidence rate of malignancy-triggered HLH (M-HLH) at our institution was 0.6% (8 of 1337). However, when the subjects were limited to leukemia and malignant lymphoma patients, the incidence rate of M-HLH was 1.9% (8 of 412).

### 3.2. Infectious Trigger and Genetic Profile

Infection screening of bacteria, fungus, and virus was performed via culture study, antigenemia assay, enzyme immunoassay (EIA), and polymerase chain reaction (PCR). Patients #2, #3, #4, #5, and #6 were positive in EBV PCR with titers of 122, 980, below 219, 614, and 13,377,547 copies/mL, respectively. The immunohistochemical staining of EBV-encoded RNA (EBER) was positive solely on the bone marrow exam of Patient #6. The respiratory virus PCR panel revealed a coronavirus OC43 infection in Patient #5 and a urinary tract infection by *Pseudomonas aeruginosa* was suspected in Patient #8. No possible pathogen was found in other patients.

A genetic study was performed in 4 patients (Patients #2, #5, #6, and #8). No mutation was detected in Patients #2 and #8. A total of 2 variants of uncertain clinical significance (VUS) were found to be heterozygous for UNC13D in Patient #5 by gene panel for familial HLH. A single VUS was noted as heterozygote for PRF1 in Patient #6. There was no mutation for SH2D1A and BIRC4 tested considering the X-linked proliferative disorder in Patient #6.

### 3.3. Clinical Course

The initial presentation and clinical outcome for each patient are summarized in Table 2. All of the patients had persistent fever, hypertriglyceridemia or hypofibrinogenemia, and hyperferritinemia. Splenomegaly (n = 7) and cytopenia in more than 2 lineages (n = 4) were found. Soluble CD25 levels were measured in 4 cases (Patient #4, #5, #6, and #8; 75.0%), and 3 of them were over 2400 U/mL. Patients #2, #3, and #8 (n = 3, 37.5%) showed hyperferritinemia above 10,000 µg/L at the time of HLH diagnosis. From the initial diagnosis of HLH, the median time taken to be diagnosed as a malignancy was about 1.3 months. A total of 3 patients (Patient #2, #6, and #8) were diagnosed as underlying malignancy far later than diagnosis of M-HLH. Their initial results of bone marrow exams were not compatible with hematologic malignancy even though follow-up results showed definite malignancy.

**Figure 1:**
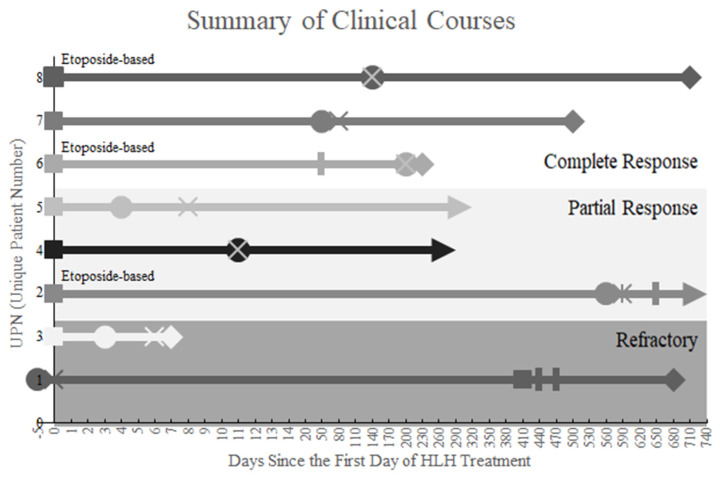
The summary of clinical courses.

The summary of clinical courses is shown in Figure 1. As the initial treatment of HLH, 3 patients (Patients #2, #6, and #8) were treated with an etoposide-based HLH treatment protocol, and 4 patients (Patients #3, #4, #5, and #7) were treated only with immunosuppressants. Patient #1 is the only patient treated initially with chemotherapy for underlying malignancy prior to HLH-specific therapy.

The summary of clinical courses is presented with several remarks indicating the milestones of treatment courses: squares (■), initiation of initial hemophagocytic lymphohistiocytosis (HLH) treatment; circles (●), diagnosis of malignancy; crosses (ⅹ), initiation of chemotherapy for underlying malignancy; bars (▐, **│**), relapse of HLH; diamonds (♦), death; and arrowheads (►), survival. Patients #2, #6, and #8 had undergone an etoposide-based HLH treatment. Each treatment outcome of HLH-specific therapy was presented on white or gray-colored zones differently saturated.

Chemotherapy for malignancy in Patient #1 was attempted at first, but was later withheld due to the exacerbation of multiple organ dysfunction caused by HLH. Although steroids were administered to relieve HLH, she recurrently suffered from infection during the period of chemotherapy, leading to a flare-up of HLH. Malignancy also progressed, and combined chemotherapy, including 100 mg/m^2^ etoposide, was initiated. It was two-thirds of the amount used according to the HLH-2004 protocol. Even with the prolonged use of immunosuppressants due to the 2nd relapse, which was 43 days after the initiation of chemotherapy, there was a 3rd relapse after 40 days more.

Patient #3 had undergone a previous immunosuppressive treatment upon diagnosis of atypical Kawasaki disease and Kikuchi disease. When the computed tomography (CT) findings were consistent with lymphoma, a biopsy was performed, and chemotherapy was then administered at the 7th day from the diagnosis of M-HLH. The HScore was 303 initially and still 311 just before chemotherapy for malignancy. The patient died of sudden progression of cerebral edema, which was suspected by CT scan, one day after starting chemotherapy.

### 3.4. Outcomes

**Figure 2:**
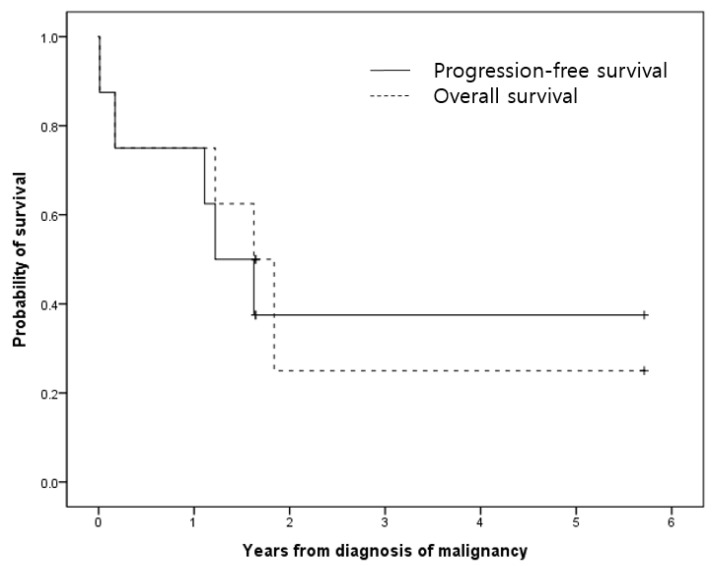
Survival analysis of patients diagnosed as malignancy-triggered HLH.

The median progression-free and overall survival from the diagnosis of malignancy was 1.2 and 1.6 years, respectively (Figure 2). Except for Patient #3, whose cause of death was acute cerebral edema, 4 patients died of other causes (progression of underlying malignancy, n = 2 (Patients #1 and #6); post transplantation complication, n = 1 (Patient #8); fungal infection, n = 1 (Patient #7)). Patient #7 and #8, who died of reasons other than disease progression, were in complete remission status at the time of death.

Survival curves were plotted against progression-free survival and overall survival by the Kaplan–Meier survival analysis. The median progression-free and overall survival from the diagnosis of malignancy was 1.2 and 1.6 years, respectively

## 4. Discussion

Immune dysregulation in HLH is alleged to be induced by impairment of granule-mediated cytotoxicity followed by hypercytokinemia. While inherited defects associated with macrophage, cytotoxic T cell, and NK cell dysfunction explain primary HLH, pathophysiology of secondary HLH is complicated with co-triggers [8,9]. The diagnostic criteria is constituted mostly with nonspecific variables and pathologic findings for HLH are not conclusive. Hemophagocytosis in bone marrow aspirates showed relatively lower specificity in establishing the diagnosis of HLH [10,11]. Because of the ambiguity of diagnosis, the incidence might be greater than previously noted. In our study, the incidence of M-HLH in hematologic malignancy patients was 1.9%; however, in other studies, it was up to 8% [12]. Unrecognized HLH flare-ups could also contribute to some morbidity and mortality.

Heterogeneous mechanisms contribute to secondary HLH under conditions of the kind of infections, mainly of viral infection such as Epstein–Barr virus (EBV), malignancy, and rheumatic disease. Accordingly, prognostic factors and treatment responses were evaluated independently in several studies. Most studies about secondary HLH proposed better outcomes with less intensive therapy and limited use of etoposide [13]. Infection-associated HLH without evidence of malignancy exhibited promising response to steroid monotherapy in a single center study in India [14]. However, EBV-associated HLH manifested worse clinical courses with a 5 year overall survival rate of 33.3% compared to 76% in HLH with unknown causes and infection other than EBV [15]. As PTLD that is routinely treated with a reduction in immunosuppression resembles EBV-associated HLH, the careful monitoring of proliferation of EBV-infected T cell and consideration of chemotherapy were recommended [16]. Treatment options in secondary HLH from steroid only to hematopoietic stem cell transplantation (HSCT) or etoposide-based protocol are not organized and still under discussion. Along with determining the severity of HLH markers and the investigation of disease triggers, a recent study suggests genetic testing as an alternative to minimize the toxicity of therapy in critically ill patients in view of its application in long-term clinical judgement on indication of HSCT or targeted biologic therapy [8,9].

In this study, we analyzed eight pediatric patients who presented with HLH as an early symptom of malignant disease, and our findings revealed that early diagnosis and the effective treatment of HLH are necessary in advance. Malignancy-triggered HLH (M-HLH) can be developed by an excessive cytokines and persistent antigen stimulation by the tumor cells. The predisposing immunodeficiency before pathologic alteration to hematologic malignancy with independent risk factors including infection can also explain a part of pathophysiology of M-HLH as in Patients #2, #6, and #8 [17]. To prevent delay in optimal therapy for underlying malignancy, a high index of suspicion is required, especially in patients showing poor response. Confound evaluation of malignancy including repeated bone marrow exam is needed [10].

M-HLH often occurred in T cell and NK-cell lymphomas or leukemias, diffuse large B-cell lymphoma(DLBCL), and Hodgkin lymphoma (HL). The predominance of T-cell malignancies was reported as more than 50% of M-HLH cases in prior series (Table 3) [18,19,20,21,22,23,24]. This is confirmative with this study as T-cell lymphoma was most frequently diagnosed (n = 6, 75%).

M-HLH has been observed to have a worse prognosis than other secondary HLHs. Nevertheless, it is not known what we should treat earlier [5]. Immunosuppression with chemotherapy for underlying malignancy was found to be insufficient to treat HLH, as seen in Patient #1. She presented with refractory HLH, which means there was no response within 2 to 3 weeks after the initial treatment [25,26]. There are few lymphomas, including ALCL, with a chemotherapy regimen that includes etoposide, and determining how to adjust chemotherapy to target both HLH and malignancy might be a breakthrough in the field [27]. Early etoposide treatment combined with HLH-directed therapy is suggested in adults with a good prognosis [9,10].

Refractory/recurrent HLH makes it difficult to manage the chemotherapy in a timely manner for underlying malignancies [5]. However, as we witnessed in the case of Patient #3, the response should be thoroughly checked after administering an initial HLH-directed regimen. An indicator to monitor the response to HLH treatment can provide useful information to decide when to start chemotherapy. In this context, we assumed that an evaluation of the HScore could be helpful because it consists of HLH components weighed differently. A comparative study of the HScore and HLH-2004 diagnostic criteria demonstrated that the HScore was more sensitive in pediatric HLH. The diagnostic sensitivity and specificity at the optimal cutoff value at the initial presentation were up to 100% and 80%, respectively, for children [6,28]. However, the score showed limitations in M-HLH in adult cohorts [28,29,30]. Our study has limitations. As a single-center retrospective study, the number of cases was small, and the therapeutic approach for HLH was heterogeneous depending on the patient’s condition at the time. It is difficult to draw clear conclusions about which therapeutic approach is optimal. However, this study suggests that some patients with secondary HLH may have hidden malignant diseases, and shows that periodic screening for malignant diseases is necessary in relatively older patients with secondary HLH. In addition, this study emphasized the need for caution as it can lead to an unexpected worsening of HLH when there is not sufficient control of HLH before treatment for malignancy.

## 5. Conclusions

Through our experiences of M-HLH as the initial presentation preceding diagnosis of malignancy, the early detection of M-HLH with a rapid screening for malignancy is critical. After diagnosis, as we had witnessed in Patient #3, the complete control of M-HLH with a HLH-directed therapy and thorough response monitoring are necessary to improve the survival rate. As it is still obscure whether M-HLH should be treated with HLH-directed therapy at first or not, the analysis of cases that first present with M-HLH before identification of malignancy will provide treatment implications. Further research on prospective cohorts is encouraging.

## Figures and Tables

**Table 1:** HLH-2004 diagnostic criteria and definition of HScore.

HLH-2004 Diagnostic Criteria [1]			
Molecular diagnosis consistent with hemophagocytic lymphohistiocytosis (HLH) or If five out of the eight criteria listed below are met			
Fever ≥ 38.5 °C			
Splenomegaly			
Cytopenias (affecting ≥ 2 of 3 lineages in the peripheral blood)			
	- Hemoglobin < 9 g/dl (in infants < 4 weeks: hemoglobin < 10 g/dL) - Platelets < 100 × 10^9^/L - Neutrophils < 1000/uL		
Hypertriglyceridemia and/or hypofibrinogenemia			
	- Fasting triglycerides >= 3.0 mmol/L (i.e., ≥265 mg/dL) - Fibrinogen ≤ 1.5 g/L		
Hemophagocytosis in bone marrow or spleen or lymph nodes			
Low or absent NK-cell activity			
Ferritin ≥ 500 ng/mL			
Soluble CD25 (i.e., soluble IL-2 receptor) ≥ 2400 U/mL			
**The HScore [6]**			
Known underlying immunosuppression *		0 (no) or 18 (yes)	
Temperature (°C)		0 (<38.4), 33 (38.4–39.4), or 49 (>39.4)	
Organomegaly	No	0	
	hepatomegaly or splenomegaly	23	
	hepatosplenomegaly	38	
Number of cytopenias (defined as a hemoglobin level of 9.2 g/dL and/or a leukocyte count of ≤5000/mm^3^ and/or a platelet count of ≤110 × 10^9^/L		1 lineage	0
		2 lineage	24
		3 lineage	34
Ferritin (ng/mL)		0 (<2000), 35 (2000–6000), or 50 (>6000)	
Triglyceride (mmol/L)		0 (<1.5), 44 (1.5–4), or 64 (>4)	
Fibrinogen (g/L)		0 (>2.5) or 30 (≤2.5)	
Serum glutamic oxaloacetic transaminase		0 (<30) or 19 (≥30)	
Hemophagocytosis on bone marrow aspirate		0 (no) or 35 (yes)	

* Underlying immunosuppression includes condition with positive human immunodeficiency virus positive or receiving long-term immunosuppressive therapy, such as glucocorticoids, cyclosporine, or azathioprine.

**Table 2:** The initial presentation and clinical outcomes of patients.

UPN	Gender	Diagnosis	Initial Treatment	DHTM (D)	Response of HLH	Clinical Symptoms of Hemophagocytic Lymphohistiocytosis (HLH)	HScore (Initial)/(d15)/(Before CTx) *	Initial Ferritin/ sCD25	Outcome	Cause of Death
#1 ^++^	F	SpTCL	CTx	4	Ref	Fever, splenomegaly, hyperTG, BM (+), and hyperferritinemia	274/224 /258	3355	Died	Progression of malignancy
#2 ^++^	F	SpTCL	Etoposide-based	615	PR	Fever, pancytopenia, hepatosplenomegaly, hyperTG, hyperferritinemia, and hyperfibrinogenemia	321/253 /(BM-)	151,828	Alive, NED	
#3	F	ALCL	Dexa	4	Ref	Fever, bicytopenia, hepatosplenomegaly, and hyperTG	303/N/A/311	11,194	Died	HLH flare-up and cerebral edema
#4	F	SpTCL	Dexa, CsA	14	PR	Fever, splenomegaly, hyperTG, BM(+), hypofibrinogenemia hyperferritinemia, and high sCD25	265/184 /146	8039 /4738	Alive, NED	
#5	M	ALCL	Dexa, CsA	4	PR	Fever, splenomegaly, hyperTG, BM(+), hyperferritinemia, and high sCD25	255/263 /248	6843 />37,500	Alive, NED	
#6 ^+^	M	EBV T L	Etoposide-based	209	CR	fever, bicytopenia, splenomegaly, Hypofibrinogenemia, hyperferritinemia, and high sCD25	208/143 /174	213 /13,506	Died	Progression of malignancy
#7	M	ALL	mPd	61	CR	Bicytopenia, hepatosplenomegaly, hyperTG, and hyperferritinemia	(BM-)/110 /133	216	Died	Fungal infection
#8	F	ALL	Etoposide-based	132	CR	Fever, pancytopenia, hypofibrinogenemia, hyperTG, and hyperferritinemia	263/107 /85	12,251 /711	Died	Pneumonia and GVHD

+; relapse once, ++; relapse twice, * HScores at initial diagnosis (Initial), the 15th day (d15) of the initial HLH treatment, and before chemotherapy (CTx) were calculated. SpTCL, subcutaneous panniculitis-like T-cell lymphoma; ALCL, anaplastic large cell lymphoma; EBV T L, Systemic EBV + T-cell lymphoma of childhood; ALL, acute lymphoblastic leukemia; CsA, Cyclosporine; CTx, chemotherapy for malignancy; Dexa, dexamethasone; hyperTG, hypertriglyceridemia; (BM+), hemophagocytosis on bone marrow aspirate; sCD25, soluble CD25; (BM-), no bone marrow exam for last one month; CR, complete response; DHTM, duration from diagnosis of HLH to diagnosis of malignancy; GVHD, Graft-versus-host disease; NED, no evidence of disease; PR, partial response; Ref, refractory; UPN, unique patient number.

**Table 3:** Characteristics and outcomes reported in previous studies of malignancy-triggered HLH (M-HLH).

Study	Number of M-HLH Patients	Type of Malignancy	Outcomes
Kai Lehmberg et al., 2015, Germany [17]	21	T-NHL (n = 8), ALCL (n = 5), T-ALL (n = 3), HL (n = 3), DLBCL (n = 2), MDS (n = 2), LPD (n = 1), and B-ALL (n = 1)	6 month survival (63–67%) Median survival time 1.2 years
Veerakul et al., 2002, Thailand [18]	24 (without distinction between M-HLH and chemotherapy-related HLH)	NHL (n = 15), mainly T-cell, ALL (n = 7) MDS (n = 1), histiocytic sarcoma (n = 1), and LCH (n = 1)	2 year survival (40.9%), 5 year survival (36%)
Tiraje Celkan et al., 2009, Turkey [19]	13	ALL (n = 5), NHL (n = 2), HL (n = 2), RMS (n = 2), NBL (n = 2), and LCH (n = 1)	Overall survival (50%)
Volker Strenger et al., 2018, Austria [20]	2	ALCL (n = 1) and GCT (n = 1)	Overall survival (50%) (Died after 23 days from diagnosis)
Hua Pan et al., 2019, China [21]	22 (without distinction between M-HLH and chemotherapy-related HLH)	AML (n = 8), B-ALL (n = 5), T-ALL (n = 4), NHL (n = 3), and HL (n = 2)	Overall survival (46.2%) Mean survival time (26.9 ± 3.82 months)
Zhizhuo Huang et al., 2020, China [22]	26	T-NHL (n = 6), AML(n = 4), LPD (n = 5), MDS (n = 2), ALL, T-ALL(n = 2), B-ALL (n = 2), ALCL (n = 1), BL (n = 1), and unknown (n = 3)	2 year survival (43.1%) (out of 27 patients including a patient with chemotherapy-related HLH)
Amitabh Singh et al., 2016, India [23]	5	AML (n = 2), T-ALL (n = 1), B-ALL (n = 1), and HL (n = 1)	Overall survival (60%) (Died within 2 weeks from the initiation of steroids)
Tekin Aksu et al., 2020, Turkey [24]	2	HL (n = 1) and ALCL (n = 1)	Overall survival (100%)

Abbreviations: T-NHL, T-cell non-Hodgkin lymphoma; ALCL, anaplastic large cell lymphoma; T-ALL, T-cell acute lymphoblastic leukemia; HL, Hodgkin lymphoma; DLBCL, diffuse large B-cell lymphoma; MDS, myelodysplastic syndrome; LPD, lymphoproliferative disorder; B-ALL, B-cell acute lymphoblastic leukemia; NHL, Non-Hodgkin lymphoma; LCH, Langerhans cell histiocytosis; RMS, rhabdomyosarcoma; NBL, neuroblastoma; GCT, germ cell tumor; BL, Burkitt lymphoma.

## Data Availability

The data presented in this study are available from the corresponding author on a reasonable request.
